# Development and Clinical Validation of a Novel 4-Gene Prognostic Signature Predicting Survival in Colorectal Cancer

**DOI:** 10.3389/fonc.2020.00595

**Published:** 2020-05-20

**Authors:** Yihang Yuan, Ji Chen, Jue Wang, Ming Xu, Yunpeng Zhang, Peng Sun, Leilei Liang

**Affiliations:** Department of General Surgery, Tongren Hospital, Shanghai Jiao Tong University School of Medicine, Shanghai, China

**Keywords:** energy metabolism, colon cancer, genes, prognosis, survival

## Abstract

In this study, we collected genes related to energy metabolism, used gene expression data from public databases to classify molecular subtypes of colon cancer (COAD) based on the genes related to energy metabolism, and further evaluated the relationships between the molecular subtypes and prognosis and clinical characteristics. Differential expression analysis of the molecular subtypes yielded 1948 differentially expressed genes (DEGs), whose functions were closely related to the occurrence and development of cancer. Based on the DEGs, we constructed a 4-gene prognostic risk model and identified the high expression of FOXD4, ENPEP, HOXC6, and ALOX15B as a risk factor associated with a high risk of developing COAD. The 4-gene signature has strong robustness and a stable predictive performance in datasets from different platforms not only in patients with early COAD but also in all patients with colon cancer. The enriched pathways of the 4-gene signature in the high- and low-risk groups obtained by GSEA were significantly related to the occurrence and development of colon cancer. Moreover, the results of qPCR, immunohistochemistry staining and Western blot assay revealed that FOXD4, ENPEP, HOXC6, and ALOX15B are over expressed in CRC tissues and cells. These results suggesting that the signature could potentially be used as a prognostic marker for clinical diagnosis.

## Introduction

Colon cancer is the fourth most common cancer worldwide and the third leading cause of cancer death (1). In 2018, it was estimated that 97,220 new cases of colon cancer would be diagnosed. In the same year, it is estimated that 50,630 people would die from colon and rectal cancers (2). The rates of colon cancer have been falling: the incidence per 100,000 population decreased from 60.5 in 1976 to 46.4 in 2005 and to 40.7 from 2009 to 2013 (3, 4). In addition, from 1990 to 2007, colon cancer mortality decreased by nearly 35% (5). However, the annual death rate and number of deaths from colon cancer remain high, and research on colon cancer and the development of treatment strategies for colon cancer remain important.

One of the hallmarks of cancer is the change in cell metabolism (6). Recent studies have shown that metabolic compartmentalization and heterogeneity exist in tumors. Autophagy in cancer (that is, when the cell digests organelles to use catabolises for metabolism) is now considered to be the driving force of tumorigenesis and cancer progression (7). It is well-known that cancer cells utilize both conventional oxidative metabolism and glycolytic anaerobic metabolism. Energy metabolism is the basis of tumor cell proliferation and invasion. This metabolism leads to epigenetic and genetic changes, along with the emergence of a variety of new cell phenotypes that enhance the proliferation and invasiveness of cancer cells. An in-depth understanding of these metabolic changes in cancer cells may lead to the development of new therapeutic strategies that, when combined with existing cancer therapies, may improve efficacy and overcome drug resistance (8). In recent years, large-scale multi-group analysis has provided us with the opportunity to search for potential prognostic markers of cancer through data mining analysis. For example, Kim et al. showed that the PAC-5 gene expression signature could predict the prognosis of patients with pancreatic adenocarcinoma through data mining (9). Xu et al. predicted a 15-gene signature related to the prognosis and recurrence of colon cancer using the GEO and TCGA databases (10). However, research on genes that predict colon cancer recurrence remains limited, and further research is needed.

In this study, genes related to energy metabolism were collected, and gene expression data from public databases such as TCGA and GEO were used to classify subtypes of colon cancer (COAD) based on the genes related to energy metabolism. The relationships between the molecular subtypes and prognosis and clinical characteristics of patients were further evaluated. The prognostic risk model constructed with the differentially expressed genes among the COAD subtypes can better evaluate the prognosis of COAD samples. Furthermore, the GEO gene expression data set was used to further verify the good performance of the prognostic risk model.

## Materials and Methods

### Data Download

We used the TCGA GDC API to download the latest clinical follow-up information. The download date was 2019.1.10, and Supplementary Table 1 contains a total of 231 cases of stage I/II RNA-Seq data samples, with expression data in Supplementary Table 2. The MINiML format GSE39582 chip expression data was downloaded from NCBI. GSE39582 contains 573 samples with clinical characteristics. The related data are shown in Supplementary Table 3, and expression data are shown in Supplementary Table 4.

### Data Pre-processing

#### TCGA Data Pre-processing

The RNA-seq data of 371 samples were pre-processed in the following steps:

1. Samples without clinical data and with OS <1 month were removed.
2. Normal tissue sample data were removed.
3. Genes with FPKM of 0 in half of the samples were removed.
4. The expression profiles of genes related to energy metabolism were maintained.

#### GEO Data Pre-processing

GSE39582 data were pre-processed in the following steps:

1. Normal tissue sample data were removed.
2. Samples with OS <1 month were removed.
3. The mapping of chip probes to the human gene SYMBOL was performed using the Bioconductor package.
4. The expression profiles of genes related to energy metabolism were maintained.

### Use of the NMF Algorithm to Identify Molecular Subtypes

A non-negative matrix factorization (NMF) clustering algorithm was used to cluster the COAD samples. For the NMF method, the standard “brunet” option was selected and 50 iterations were carried out. The number of clusters k was set as 2 to 10, the average contour width of the common member matrix was determined through the R package “NMF,” and the minimum member of each subclass was set as 10. The cophenetic, dispersion and silhouette indicators were used to determine the optimal clustering number, the optimal clustering number selected was 4. Further, we calculated the proportion of gene mutations in the different subtypes and selected the top 20 genes with the highest mutation rates in each subtype.

### Comparison of the Clinical Characteristics of the Molecular Subtypes

The gene sets related to energy metabolism were used to cluster COAD into four subtypes, and the differences in the clinical characteristics including T stage, stage, sex and age of the four subtypes were compared. Further, the Tumor Immune Estimation Resource (TIMER) was used to compare the immune scores of the four subtypes.

### Identification and Functional Analysis of Differentially Expressed Genes

DESeq2 was used to calculate the differentially expressed genes (DEGs) between the C2 and C1/C3/C4 molecular subtypes with the best prognosis. A total of 2459 differentially expressed genes (padj. < 0.05 and |log2FC|>1) shared by the two groups were identified, and a total of 1,948 genes were removed due to redundancy. Further, KEGG and GO functional enrichment analysis was carried out on the 1948 DEGs through the R package clusterProfiler, and the selection threshold was *p* < 0.05.

### Risk Model Construction in the Training Set

After pre-processing the stage I/II TCGA samples, randomly allocate 50% of the 231 samples as the training set for model building. To avoid deviation affecting the stability of the subsequent modeling, we randomly generated 100 times of all samples in advance with repeated sampling to ensure that the age, stage and TNM staging distributions of the random samples were in agreement with those of all the samples. A univariate Cox proportional risk regression model was performed for each DEG with survival data. The coxph function in the survival R package was used, and *p* < 0.01 was selected as the threshold. Finally, there were 26 genes with significant differences in prognosis. We selected 26 genes with significant clinical variables and carried out feature selection using the randomForestSRC software package. We also used the randomSurvivalForest algorithm to rank the importance of prognostic-related genes (nrep = 100, which indicates that the number of iterations in the Monte Carlo simulation was 100; and nstep = 5, which indicates that the number of steps forward was 5). We identified genes with a relative importance >0.65 as the final signature.

### Use of Multivariate Regression to Establish a Prognostic Model

Further, we performed multivariate regression analysis on the four genes obtained from the random forest algorithm. The importance and relative importance of the coefficients, HRs, confidence intervals, Z scores and out-of-bag estimates of the four genes were determined. Then, a 4-gene signature was established, and the model was as follows:

$$\begin{array}{r} \text{RiskScore}4=0.893\;*\text{ expFOXD}4+0.234\;*\text{ expENPEP}\\ +0.173\;*\text{ expHOXC}6+0.061\;*\text{ expALOX}15\text{B}. \end{array}$$

### ROC Analysis of the Risk Model

The RiskScore of each sample is calculated according to the expression level of the sample, and the RiskScore distribution of the sample is drawn. Further, the R software package timeROC was used to perform ROC analysis of the RiskScore prognosis classification to analyse the classification efficiency of the prognosis predictions for 1 year, 3 years and 5 years.

### Internal and External Data Sets Verify the Robustness of the 4-Gene Signature

To determine the robustness of the model, the same model and the same coefficients as those in the training set were used in the internal validation set. First, for the stage I/II validation set samples, the expression level of each sample was calculated separately. For the risk score, we used the R package timeROC to perform ROC analysis to analyse the RiskScore prognostic classification efficacy for 1, 2, and 3 years. Furthermore, we used the same model and the same coefficients as those in the training set in all colon cancer TCGA samples. We also calculated the RiskScore of each sample according to the expression level of 427 samples, used the R software package timeROC to carry out ROC analysis on the prognosis classification of the RiskScore and analyzed the classification efficiency of the 1, 2, and 3-year prognosis predictions.

To determine the robustness of the model, we used the same model and the same coefficients as those in the training set in GEO data sets (same steps as before).

### Risk Model and Clinical Characteristic Analysis

To assess the relationship between the prediction accuracy of the risk model and the prediction accuracy of clinical features, we analyzed the prediction relationship between age, sex, stage, T stage, and RiskScore from the perspectives of single and multiple factors and then constructed a nomogram model with clinical features including age, sex, stage, T stage, and RiskScore.

### GSEA-Enriched Pathways in the High-Risk Group and the Low-Risk Group

GSEA was used on 231 TCGA training cases to analyse the significantly enriched pathways in the high-risk group and the low-risk group. The selected gene set was c2.cp.Kegg. The GSEA input file contains expression profile data standardized by the TCGA training set as well as the sample labels of the 4-gene signature. The sample labels mark the sample as belonging to the high-risk group or the low-risk group. The threshold of the enriched pathways was *p* < 0.05.

### Sample Collection

CRC and adjacent tissues were collected from 30 patients (all participants were older than 16 years, Minimum:46, Maximum:85, SD:11.43, mean:62.3)immediately placed in liquid nitrogen, and preserved at −80°C. None of the colorectal cancer patients received preoperative anti-tumor therapies. Patients and their families in this study have been fully informed and informed consent was obtained from the participants. This study was approved by the Ethics Committee of Shanghai Tongren Hospital.

### Cell Culture

Human normal colorectal epithelial cell line (NCM460) and CRC cell line, including SW480 and SW620, cells were obtained from Shanghai Cell Bank of the Chinese Academy of Sciences (Shanghai, China). NCM460, SW480, and SW620 cells were cultured in 90% DMEM (Gibco) supplemented with antibiotics (1 × penicillin/streptomycin100 U/ml, Gibco) and 10% heat-inactivated fetal bovine serum (FBS) (Gibco, Grand Island, NY, USA). The cells were incubated at 37°C in a humidified and 5% CO_2_ incubator.

### RNA Isolation and PCR Analysis

Total RNA from the CRC tissue specimens was extracted by TRIzol reagent (Invitrogen, Thermo Scientific, Shanghai, China), and RNA was reverse transcribed into cDNA with the QuantiTect Reverse Transcription Kit (QIAGEN, Valencia, CA, USA). Real-time PCR analyses were quantified with SYBR-Green (Takara, Otsu, Shiga, Japan), and the levels were normalized to the level of GAPDH.

### Immunohistochemical Staining

Paraffin-embedded tissues were immunostained for FOXD4, ENPEP, HOXC6, and ALOX15B proteins. The slides were dried, deparaffinized and rehydrated. Then, the slides were immersed in 3% hydrogen peroxide and labeled with antibodies overnight. Anti-FOXD4 (1:200), anti-ENPEP (1:200), anti-HOXC6 (1:200) and anti-ALOX15B (1:200) were purchased from Abcam (Cambridge, UK). Image-Pro Plus 6.0 Software (Media Cybernetics, USA) was used for the protein expression analysis.

### Western Blot Assay and Antibodies

Western blot analysis was performed as previously described (11). Antibodies against FOXD4 (dilution 1:3000, Abcam), ENPEP (dilution 1:3000, Abcam), HOXC6 (dilution 1:3000, Abcam,) and ALOX15B (dilution 1:3000, Abcam) were used. The bands were visualized with ECL reagent (Thermo Fisher Scientific, USA) and GAPDH (dilution 1:3000, Abcam) was used as the loading control.

### Statistical Analysis

Student's *t-*test was used to examine the differences between groups. A value of *p* < 0.05 was regarded as statistically significant. All calculations were performed using SPSS software version 13.0.

## Results

### Source of the Genes Related to Energy Metabolism

Human metabolism-related pathways were downloaded from Reactome (https://reactome.org/), and a total of 594 genes related to energy metabolism were collated from the 11 metabolic pathways, as shown in Table 1.

**Table 1:** Pathways related to energy metabolism in the Reactome database.

**Metabolic pathways from Reactome**	**PathwayID**	**Gene count**
Biological oxidations	R-HSA-211859	221
Metabolism of carbohydrates	R-HSA-71387	292
Mitochondrial Fatty Acid Beta-Oxidation	R-HSA-77289	38
Glycogen synthesis	R-HSA-3322077	16
Glycogen metabolism	R-HSA-8982491	27
Glucose metabolism	R-HSA-70326	92
Glycogen breakdown (glycogenolysis)	R-HSA-70221	15
Glycolysis	R-HSA-70171	72
Pyruvate metabolism	R-HSA-70268	31
Pyruvate metabolism and Citric Acid (TCA) cycle	R-HSA-71406	55
Citric acid cycle (TCA cycle)	R-HSA-71403	22
Sum	881(unique:594)	

### Data Pre-processing

The statistical information of the pre-processed dataset is shown in Table 2.

**Table 2:** Clinical information of the two data sets after pre-processing.

**Characteristic**		**TCGA training datasets (*n* = 115)**	**TCGA validation datasets (*n* = 116)**	**GSE39582 (*n* = 573)**
Age(years)	< =60	28	30	245
	>60	87	86	328
Survival Status	Living	100	102	150
	Dead	15	14	66
Gender	Female	56	49	256
	Male	59	67	317
pathologic_T	T 1	3	5	12
	T 2	25	35	48
	T 3	82	70	372
	T 4	5	5	117
pathologic_N	N 0	115	116	307
	N 1	0	0	134
	N 2	0	0	100
	N 3	0	0	6
pathologic_M	M 0	106	103	491
	M 1	0	0	60
	M X	8	12	0
Tumor stage	Stage I	28	41	37
	Stage II	87	75	265
	Stage III	0	0	208
	StagIV	0	0	59

### Molecular Typing Based on Energy Metabolism Genes

**Figure 1:**
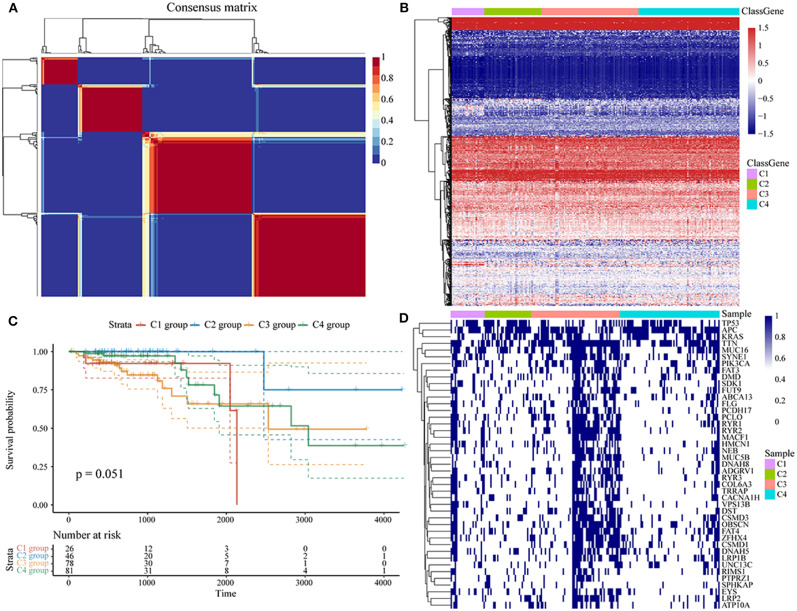
**(A)** Consensus map of NMF clustering; **(B)** heat map of energy metabolism-related gene expression of the molecular subtypes; **(C)** OS prognosis and survival curves of the molecular subtypes; **(D)** the mutation landscape of the top 20 genes with the highest mutations in each subtype in each sample.

Molecular subtypes were identified using the NMF algorithm. According to the cophenetic, dispersion and silhouette indicators, the optimal clustering number of 4 was selected (Figure 1A, Supplementary Figure 1). The energy metabolism-related gene expression of the four subclasses (Figure 1B) can be seen from the diagram as parts with differences in gene expression. We further analyzed the prognosis of the four groups. The results show that C1 and C4 had the worst prognosis, C2 had a better prognosis, and the 4 subtypes had significantly different prognoses, as seen in Figure 1C (log-rank *p* = 0.052). Further, we counted the proportions of genetic mutations according to the different subtypes, selected the top 20 genes from each subtype with the highest percentages of mutations, and obtained 45 gene mutations, suggesting that the gene mutations with the highest frequency are largely different among the four subtypes. The 45 gene mutations of each subtype are visually displayed in Figure 1D, which shows various subtypes according to frequency differences of the 45 gene mutations. Each subtype of samples has certain differences in gene mutation frequency.

**Figure 2:**
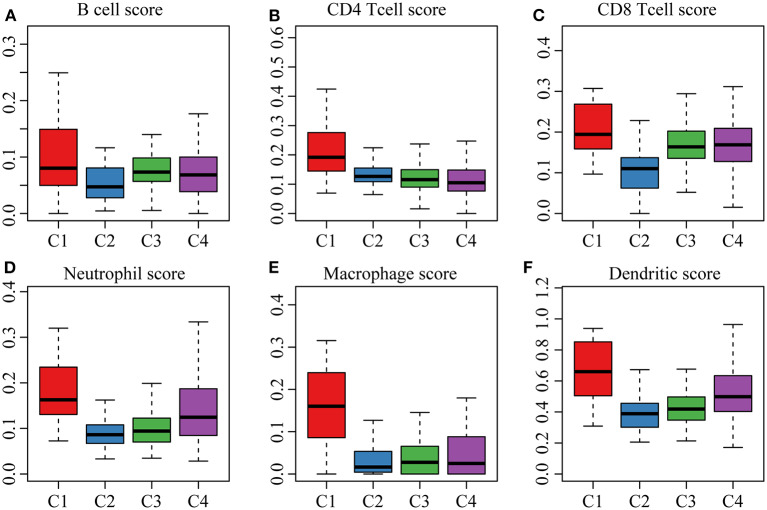
**(A,B)** cell scores of the molecular subtypes; **(B)** CD4 cell scores of the molecular subtypes; **(C)** CD8 cell scores of the molecular subtypes; **(D)** neutrophil scores of the molecular subtypes; **(E)** macrophage scores of the molecular subtypes; **(F)** dendritic cell scores of the molecular subtypes.

The clinical characteristics of molecular subtypes were then compared (Table 3). The gene sets related to energy metabolism were used to cluster COAD into four subtypes. We compared the differences in the clinical characteristics of T stage, stage, sex and age among the four subtypes, and no significant differences were observed (Supplementary Figure 2). Further, using the TIMER (tumor immune estimation resource) tool, we compared the immune scores of the four subtypes. The scores of five immune cells (B cells, CD8 T cells, neutrophils, macrophages and dendritic cells) found in the C2 subtype were all lower than those in the C1, C3, and C4 subtypes (Figure 2), while the scores of the immune cells of the C1 samples were all significantly higher than those of the other subtypes. This finding may indicate that there is a complicated relationship between immune invasion and prognosis in COAD patients. The score data of the six immune cells of all samples are shown in Supplementary Table 5.

**Table 3:** Clinical information statistics of the molecular subtypes.

**Clinical features**	**C1**	**C2**	**C3**	**C4**
**Event**				
Alive	24	45	64	71
Dead	2	1	13	10
**T**				
T1	0	2	3	3
T2	2	13	21	24
T3	23	29	50	50
T4	1	2	4	3
**Stage**				
I	2	15	24	28
II	24	31	54	53
**Gender**				
Female	9	22	36	38
Male	17	24	42	43
**Age**				
< =60	9	14	16	19
>60	17	32	62	62

### Analysis of Differentially Expressed Genes Among the Subtypes

**Figure 3:**
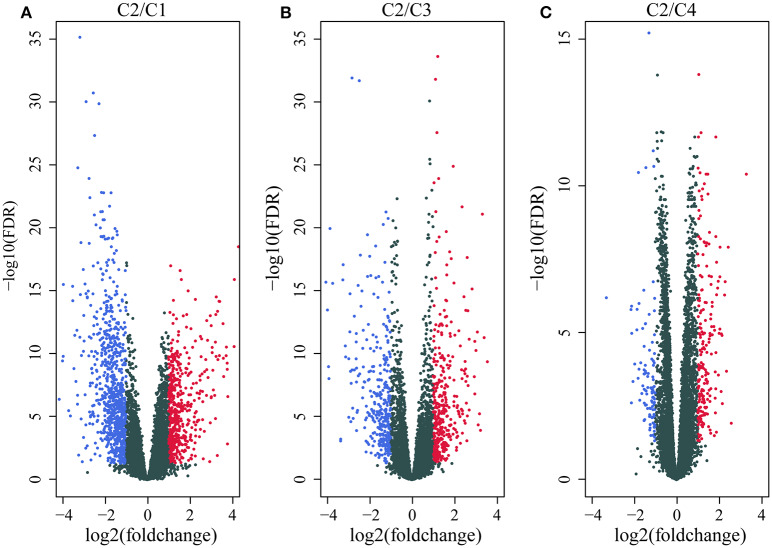
**(A)** Volcano map of the differentially expressed genes between the C2 and C1 subtypes; **(B)** volcano map of the differentially expressed genes between the C2 and C3 subtypes; **(C)** volcano map of the differentially expressed genes between the C2 and C4 subtypes.

We first identified the differentially expressed genes. DESeq2 was used to calculate the differentially expressed genes (DEGs) between the C2 and C1/C3/C4 molecular subtypes with the best prognosis. A total of 2459 differentially expressed genes (padj. < 0.05 and |log2FC|>1) shared by the two groups were identified, and a total of 1,948 genes were removed for redundancy. A volcano plot of the upregulated and downregulated differentially expressed genes (C2~C1, C2~C3, and C2~C4) is shown in Figures 3A–C. As shown in the figure, the differentially downregulated genes between the C2 and C1 subtypes are redundant and upregulated, and of the main differentially expressed genes between the C2 and C3 subtypes and the C2 and C4 subtypes, less are upregulated and more are downregulated; the differentially expressed genes are shown in Supplementary Table 6.

**Figure 4:**
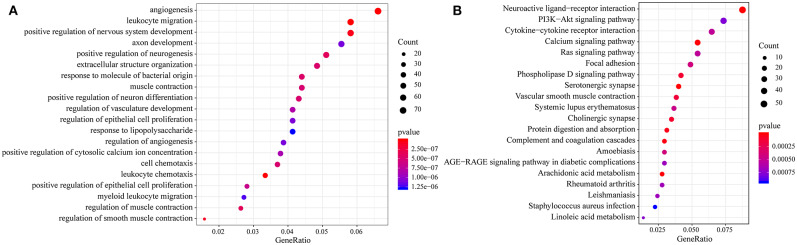
**(A)** Results of GO enrichment of the top 20 differentially expressed genes; **(B)** KEGG enrichment of the top 20 genes.

Then, we performed functional analysis of the differentially expressed genes. Further, we conducted KEGG and GO functional enrichment analysis on these 1948 DEGs with the R package clusterProfiler and the selected threshold of p <0.05. The results are shown in Supplementary Tables 7, 8. The DEGs were enriched in 69 KEGG pathways and 1109 GO terms. The top 20 GO terms are shown in Figure 4A, including angiogenesis, leukocyte migration and positive regulation of epithelial cell proliferation. The top 20 enriched pathways are shown in Figure 4B, and the most significant pathways were the PI3K-Akt signaling pathway, calcium signaling pathway, AGE-RAGE signaling pathway in diabetic complications and other cancer-related pathways.

### Construction of a Prognostic Risk Model Based on the DEGs

**Figure 5:**
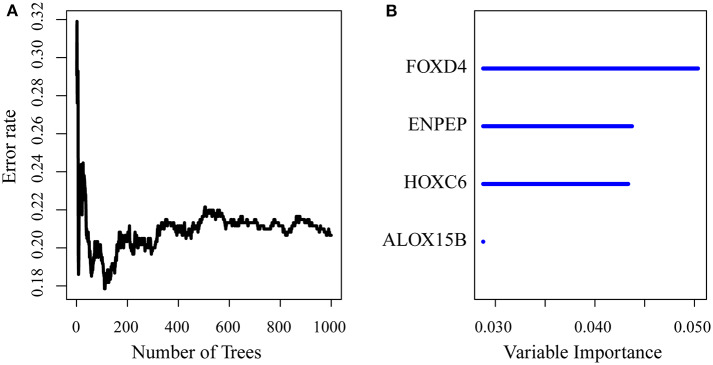
**(A)** Error rate for the data as a function of the classification tree; **(B)** out-of-bag importance values for the predictors.

First, the risk model was constructed with the training set. According to the above method, we obtained the training set sample (as shown in Table 2), which contained a total of 26 genes with significant prognostic differences (Supplementary Table 10). Further, the randomForestSRC R software package was used for feature selection. We identified genes with a relative importance <0.65 as the final signature. Figure 5A shows the relationship between the error rate and the number of classification trees, and Figure 5B shows the order of the out-of-bag importance of the first four genes.

Then, we established a multivariate regression model for prognosis. We performed multivariate regression analysis on the four genes obtained from the random forest algorithm, and the importance and relative importance of the coefficients, HRs, confidence intervals, Z scores and out-of-bag estimates of the four genes are shown in Table 4.Then, a 4-gene signature was established, and the model was as follows:

$$\begin{array}{r} \text{RiskScore}7=0.893\;*\text{ expFOXD}4+0.234\;*\text{ expENPEP}+\\ 0.173\;*\text{ expHOXC}6+0.061\;*\text{ expALOX}15\text{B} \end{array}$$

**Table 4:** Four genes significantly associated with overall survival in the training set patients.

**Symbol**	**Coef**	**HR**	**Lower 95% CI**	**Upper 95% CI**	**Z-score**	**Importance**	**Relative importance**
FOXD4	0.8934	2.443	1.154	5.175	2.333	0.0147	1
ENPEP	0.23431	1.264	1.003	1.593	1.985	0.0126	0.8567
HOXC6	0.17337	1.189	1.009	1.402	2.063	0.0116	0.7913
ALOX15B	0.06069	1.063	0.907	1.245	0.751	0.0098	0.6657

**Figure 6:**
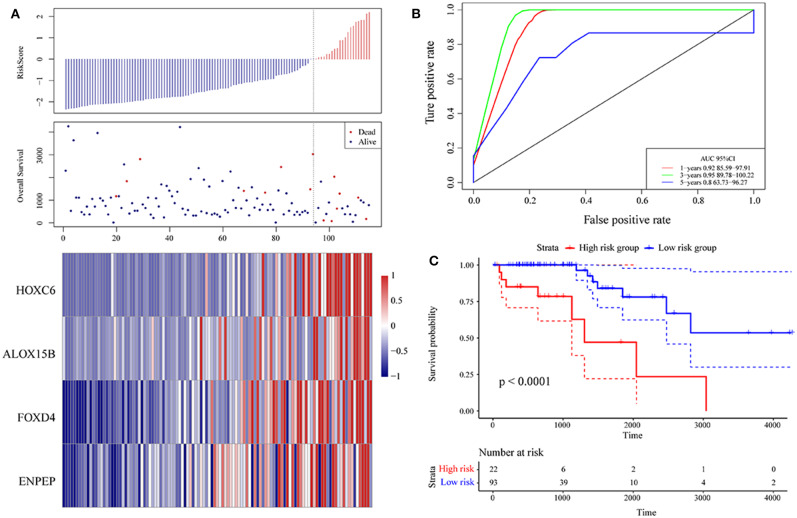
**(A)** Risk score, survival time, survival state and expression of the 4 genes in the training set; **(B)** ROC curve and AUC of the 4-gene signature classification; **(C)** distribution of KM survival curves of the 4-gene signature in the training set.

Then, ROC analysis of the risk model was carried out. The expression level of the risk score of each sample is calculated, and the RiskScore distribution of samples is drawn. As shown in Figure 6A, compared with low risk scores, high risk scores can be seen from the diagram of OS, suggesting that the samples with high RiskScores have worse prognosis. In different samples, the change in the gene expression of FOXD4, ENPEP, HOXC6, and ALOX15B increased the risk value and therefore, the high expression of these genes was identified as a risk factor. Further, we used the R software package timeROC to conduct ROC analysis on the prognosis classification of the RiskScore. We analyzed the classification efficiency of the prognosis prediction for 1, 3, and 5 years, as shown in Figure 6B. It can be seen that the model has a high AUC offline area, the AUC is above 0.8 and the AUC offline area for the 3-year prediction reaches 0.95. We calculated the Gordon index as the cut-off (2.563985) for the sample group based on the AUC value of the 3-year prediction and found that the samples were clearly divided into high-risk and low-risk groups. The KM curves were drawn (as shown in Figure 6C), and a markedly significant difference of *p* < 0.0001 was observed between the groups.

### Internal and External Data Sets Verify the Robustness of the 4-Gene Signature

**Figure 7:**
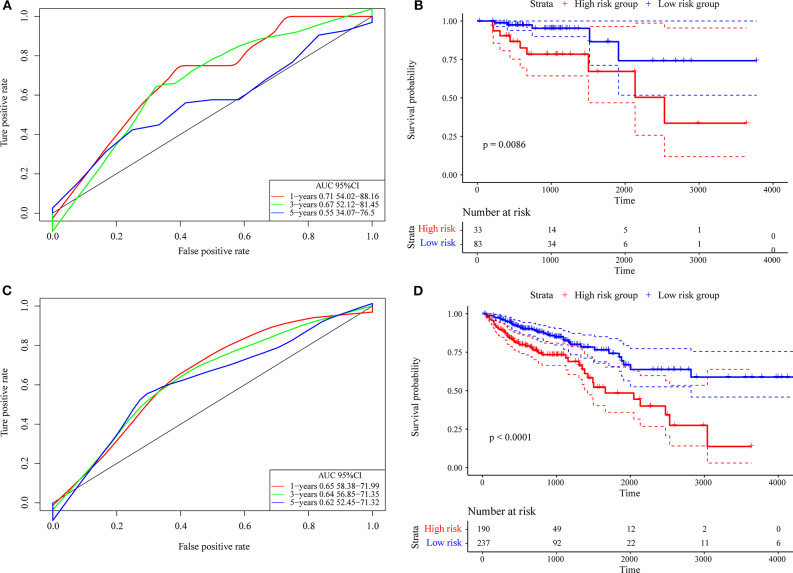
**(A)** The internal validation set contains only stage I/II samples for the 4-gene signature classification ROC curve and AUC; **(B)** stage I/II samples for the 4-gene signature KM survival curve; **(C)** the internal validation set contains stage I, II/I, and II/IV samples for the 4-gene signature classification ROC curve and AUC; **(D)** stage I, II/I, and II/IV samples for the 4-gene signature KM survival curve.

First, we used the internal data set to verify the robustness of the 4-gene signature. As shown in Figure 7A, the model has a high AUC offline area for the 1- and 3-year predictions, while the AUC for the 5-year prediction is above 0.65. Similarly, we calculated the cut-off value (1.751732) with the Gordon index for the sample groups based on the AUC value of the 1-year prediction, divided the samples into high-risk and low-risk groups and drew KM curves, as shown in Figure 7B. It can be seen that there is a markedly significant difference between them, with *p* = 0.0086.

Further, we used the same model and the same coefficients as those in the training set to calculate the risk score in 427 colon cancer TCGA samples. The results are shown in Figure 7C, which shows that the AUC line area of the model is above 0.6. Similarly, we calculated the cut-off value (1.232182) with the Gordon index for the sample groups based on the AUC value, divided the samples into high-risk and low-risk groups and drew the KM curves, as shown in Figure 7D. It can be seen that there is a markedly significant difference (*p* < 0.0001) between the groups.

**Figure 8:**
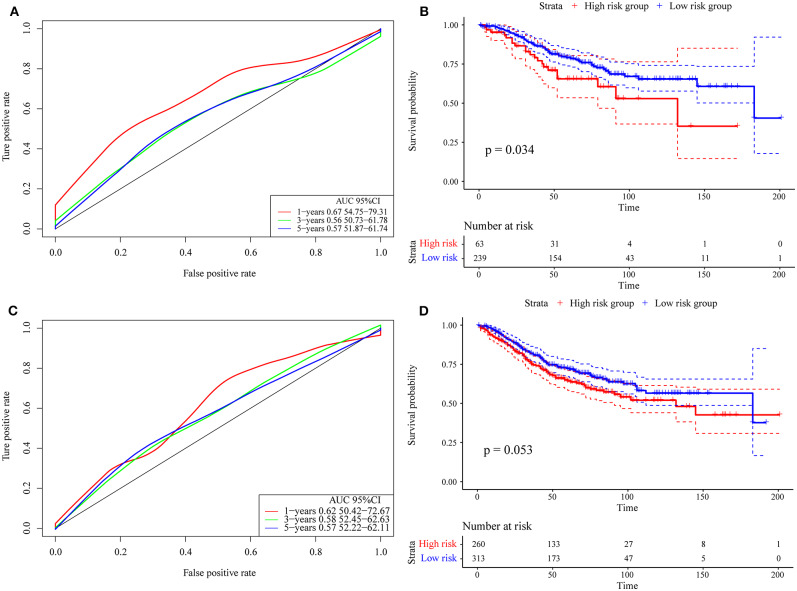
**(A)** Risk score, survival time, survival state and expression of the 4 genes in the external verification set; **(B)** ROC curve and AUC of the 4-gene signature classification; **(C)** distribution of KM survival curves of the 4-gene signature in the training set. **(D)** Stage I, II/I, and II/IV samples for the 4-gene signature KM survival curve.

Finally, we verified the robustness of the 4-gene signature with an external data set. We adopted the same model and the same coefficients as those in the training set in a set of GEO data sets. As shown in Figure 8A, the annual AUC of the model is above 0.67. Similarly, we calculated the cut-off value (5.989193) with the Gordon index for the sample groups based on the AUC value of the 1-year prediction, divided the samples into high-risk and low-risk groups and drew the KM curves, as shown in Figure 8B. It can be seen that there is a markedly significant difference between the two groups (*p* = 0.034).

Further, we used the same model and the same coefficients as those in the training set to calculate the risk score in 573 colon cancer samples. As shown in Figure 8C, the AUC offline area of the model for the one-year prediction is above 0.62. Similarly, we calculated the cut-off value (5.53358) with the Gordon index for the sample groups based on the AUC value of the one-year prediction, divided the samples into high-risk and low-risk groups and drew the KM curves, as shown in Figure 8D. It can be seen that there is a marginally significant difference between the groups (*p* = 0.053).

### Risk Model and Clinical Characteristic Analysis

To assess the relationship between the prediction accuracy of the risk model and the prediction accuracy of clinical features (Table 5), we analyzed the prediction relationship between age, sex, stage, T stage, and RiskScore from the perspectives of single factor and multiple factors. The final results are shown in Supplementary Table 9.

**Table 5:** Cox regression analysis.

**Characteristic**	***p*-value**	**HR**	**Low 95%CI**	**High 95%CI**
4-gene risk socre	2.41E-05	2.718	1.709	4.324
Age	0.004167561	1.123	1.037	1.216
Gender	0.458536522	1.492	0.518	4.297
Height	0.95664815	1.002	0.939	1.069
Weight	0.239722298	0.966	0.912	1.023
BMI	0.206292748	0.974	0.936	1.014
StageI-vs-II	0.965867387	0.967	0.207	4.508
T2-vs-T3	0.92519835	1.107	0.133	9.235

**Figure 9:**
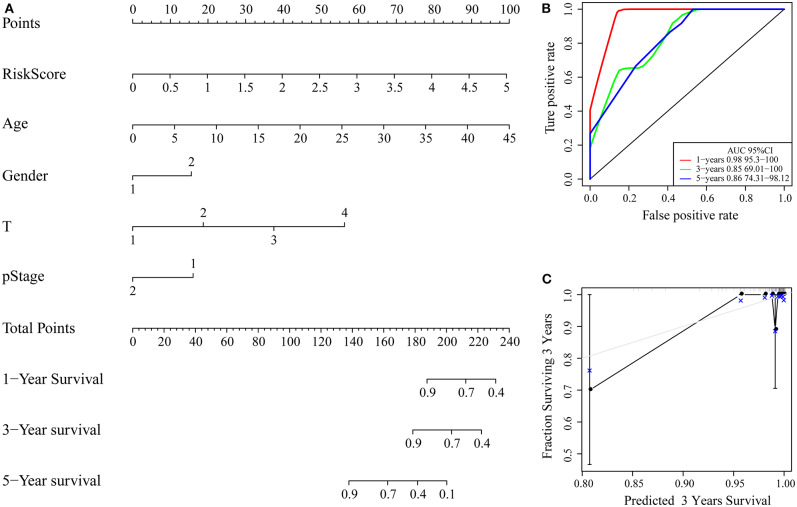
**(A)** Nomogram; **(B)** ROC curve of the nomogram; **(C)** calibration plots for predicting 3-year OS. The nomogram-predicted aim-listed probability of survival is plotted on the x-axis. The actual survival is plotted on the y-axis.

Clinical characteristics including age, sex, stage, T stage and RiskScore were used to construct a nomogram model, as shown in Figure 9A. Further, the prediction accuracy of the nomogram was assessed by ROC analysis, and the results showed that the AUCs of the nomogram for the 1, 3, and 5-year predictions were 0.98, 0.85, and 0.86, respectively (Figure 9B). Figure 9C shows the comparison between the 3-year nomogram model and the ideal model, and the results show that some indices of the 3-year nomogram model are basically consistent with those of the ideal model, indicating that the accuracy of our model is relatively high.

### GSEA Reveals the Pathways Enriched in the High-Risk Group and the Low-Risk Group

GSEA was used in 342 TCGA training cases to analyse the significantly enriched pathways in the high-risk group and the low-risk group. The selected gene set was c2.cp.Kegg. The GSEA input file contains expression profile data standardized by the TCGA training set and the sample labels of the 4-gene signature. The sample labels mark the sample as belonging to either the high-risk group or low-risk group. The threshold value of the enriched pathways was *p* < 0.05, and the obtained significantly enriched pathways are shown in Supplementary Table 10.

**Figure 10:**
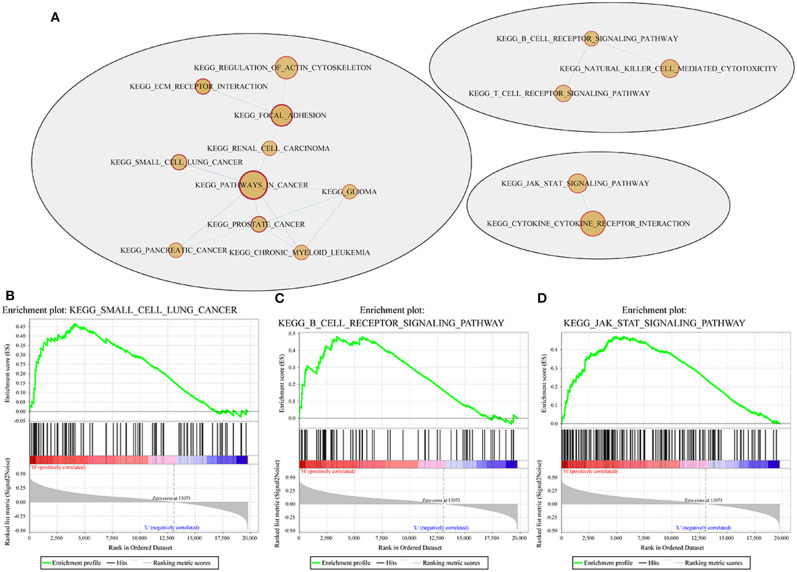
Pathways enriched in the high- and low-risk groups according to the 4-gene signature. **(A)** Network map mapped by the GSEA enrichment gene set (red represents the high-risk group); **(B–D)** results of significantly enriched pathways in the high-risk group by GESA. Enrichment scores (ES, green line) indicate the degree to which the genome is overexpressed at the top or bottom of the list of sequenced genes. The black bars represent the positions of genes belonging to the set of genes in the list of sequences included in the analysis. Positive values indicate a higher correlation with patients in the high-risk group, while negative values indicate a higher correlation with patients in the low-risk group.

For example, the B cell receptor signaling pathway, JAK STAT signaling pathway, small cell lung cancer and other pathways were significantly enriched in the high-risk group, as shown in Figure 10.

### FOXD4, ENPEP, HOXC6, and ALOX15B Expression Is High in CRC Tissues

**Figure 11:**
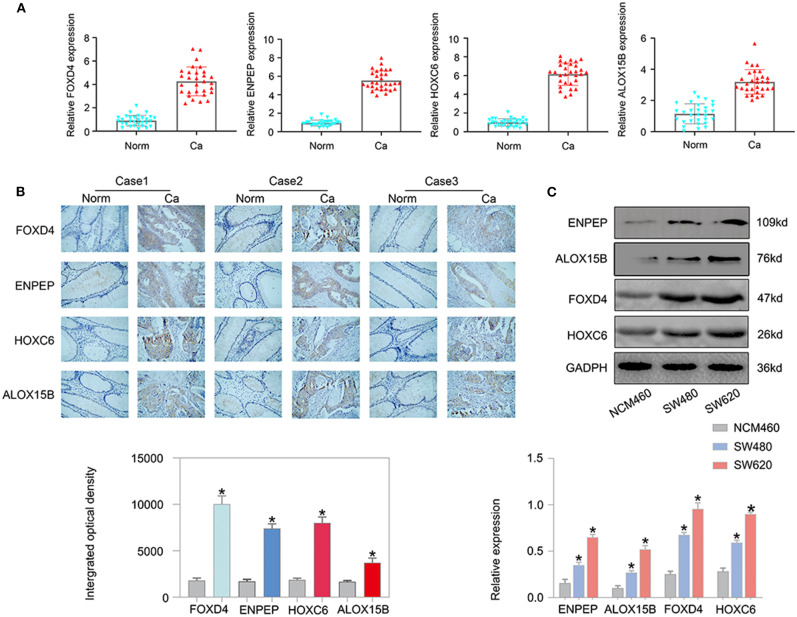
The expression of the oncogenes is up-regulated in CRC. According to the qPCR results **(A)**, FOXD4, ENPEP, HOXC6 and ALOX15B were up-regulated in CRC tissues. **(B)** Immunostaining demonstrated that FOXD4, ENPEP, HOXC6, and ALOX15B were up-regulated in CRC tissues compared with normal tissues**. (C)** The results of Western blot assay showed that the expression of FOXD4, ENPEP, HOXC6, and ALOX15B are over expressed in CRC cells. *P < 0.05.

Next, we examined the expression of the oncogenes (FOXD4, ENPEP, HOXC6, and ALOX15B) by qPCR in 30 pairs of clinical samples from CRC patients. According to the qPCR results, the oncogenes were expressed at high levels in CRC tissues (Figure 11A). Correspondingly, immunostaining analyses of the oncogenes (FOXD4, ENPEP, HOXC6, and ALOX15B) were performed in the cancerous and normal tissues, and immunostaining demonstrated that the expression of oncogenes (FOXD4, ENPEP, HOXC6, and ALOX15B) was high in the cancerous tissue (Figure 11B). The results of Western blot assay showed that the expression of FOXD4, ENPEP, HOXC6, and ALOX15B are over expressed in CRC cells (SW480 and SW620 cells) compare with NCM460 cells (Figure 11C).

## Discussion

Colon cancer is a malignant tumor with poor prognosis. Currently, non-metastatic colon cancer can be treated with surgery or adjuvant chemotherapy (12). However, chemotherapy has considerable toxicity (13–15). Energy metabolism is the basis of tumor cell proliferation and invasion, and most tumor cells show deviation from the normal energy metabolism state so that they can survive and eventually grow under challenging microenvironmental conditions (16). However, the relationship between energy metabolism genes and the prognosis of tumor cells is still unclear.

We used GEO and TCGA public gene expression data. Based on the 594 energy metabolism-related genes for early COAD classification, the samples can be classified into four subtypes, with significant differences in prognosis between the subtypes. The analysis of the expression differences between the molecular subtypes resulted in 1948 differentially expressed genes (DEGs), and the function of the DEGs are closely associated with cancer development. Based on the DEGs, we built a four-gene prognostic risk model and evaluated its validity. Based on the dataset containing 371 TCGA samples, we identified potential prognostic marker genes (FOXD4, ENPEP, HOXC6, and ALOX15B). When highly expressed, all four genes are risk factors associated with a high risk of developing colon cancer. According to previous reports, FOXD4 induces the progression of colorectal cancer by regulating the SNAI3/CDH1 axis and can be used as a marker of colorectal cancer (17). When ENPEP is silenced, the occurrence of breast cancer can be inhibited (18); moreover, both *in vitro* and *in vivo*, ENPEP silencing and impaired ENPEP activity reduce the proliferation, migration and drug resistance of colorectal cancer (19). HOXC6 is a classic cancer-related gene. In cervical cancer, enhanced HOXC6 expression leads to cervical cancer cell proliferation, cell cycle progression, colony formation anchoring and xenograft tumor growth (20). In nasopharyngeal carcinoma, HOXC6 is an independent prognostic parameter for NPC patients, and HOXC6 expression is positively correlated with the Ki-67 proliferation index (21). In prostate cancer, the upregulation of HOXC6 can not only participate in the process of PCa but also serves as an independent prognostic indicator of cancer (22). In gastric cancer, the upregulation of HOXC6 can increase the migration and invasion ability of gastric cancer cells, while the interference of HOXC6 expression can inhibit the migration and invasion of gastric cancer cells. The upregulation of HOXC6 expression can enhance MMP9 expression, while the downregulation of HOXC6 can reduce MMP9 gene expression. The increased expression of HOXC6 in gastric cancer cell lines significantly activated extracellular signals regulating kinase signal transduction and MMP9 upregulation, which promoted the migration and invasion of gastric cancer cells (23). In HCC, HOXC6 may promote the invasion of HCC by driving epithelial-mesenchymal transformation (EMT) (24). ALOX15B may promote the development of non-small cell lung cancer and female breast cancer (25, 26). Further, the Tumor Immune Estimation Resource (TIMER) was used to compare the immune scores of the four subtypes. In previous research, FOXD4, ENPEP, HOXC6, and ALOX15B are closely related to the immune system. For example, FOXD4 have been implicated in at least four familial human diseases, and differential expression may play a role in a number of other pathologies-ranging from metabolic disorders to autoimmunity (27). ENPEP is known to be associated with inflammatory or immune response that may be associated with mechanisms of major depressive disorder (28). HOXC6 is related to overall survival and intestinal immune network of the right-sided colon cancer (29). ALOX15B activity is associated with inflammation and immune regulation in the pathogenesis of inflammatory lung disorders (30). In addition to these gene markers, our study also identified significantly enriched pathways, including the B cell receptor signaling pathway, JAK STAT signaling pathway, and small cell lung cancer, which are significantly related to the occurrence and development of cancer. The JAK STAT signaling pathway has been indicated to be related to the progression and prognosis of colon cancer in a variety of studies (31–33).

Subsequently, 573 colon cancer samples from the GEO database were used as the verification set to prove that this 4-gene signature has strong robustness and a stable predictive performance in data sets from different platforms. This gene signature is not only stable in early stage colon cancer patients but also in all colon cancer patients. The pathways enriched in the high- and low-risk groups obtained by GSEA for the 4-gene signature were significantly related to the occurrence and development of colon cancer, suggesting that this signature could potentially be used as a prognostic marker for clinical diagnosis. Moreover, the results of qPCR, immunohistochemistry staining and Western blot assay revealed that FOXD4, ENPEP, HOXC6, and ALOX15B are over expressed in CRC tissues and cells.The advantage of this study is that we identified a prognostic 4-gene signature that had relatively high AUCs for 1/3/5-year survival rate predictions in the training and validation data sets. However, the study had some limitations. Such as, the 4-gene signature lacks experimental validation. Despite the high quality of RNA-seq data in TCGA, further experimental validation in *vitro* and *vivo* of these four genes in colon cancer are needed.

In summary, our study revealed a 4-gene signature associated with prognosis in colon cancer patients. The signature can be used as a potential candidate biomarker and therapeutic target for colon cancer patients.

## Data Availability Statement

Publicly available datasets were analyzed in this study. This data can be found in The Cancer Genome Atlas (TCGA) Data Portal (https://tcga-data.nci.nih.gov/tcga) and the NCBI Gene Expression Omnibus (GSE39582).

## Ethics Statement

This study was approved by the Ethics Committee of Shanghai Tongren Hospital. All subjects gave written informed consent in accordance with the Declaration of Helsinki.

## Author Contributions

LL and PS conceived this study. YY and YZ performed the experiments. JC, MX, and JW prepared the manuscript. All authors approved the final version of the manuscript.

## Conflict of Interest

The authors declare that the research was conducted in the absence of any commercial or financial relationships that could be construed as a potential conflict of interest.
