# Supplementary figures and images for: Development and Clinical Validation of a Novel 4-Gene Prognostic Signature Predicting Survival in Colorectal Cancer
Source: Front Oncol. 2020 May 20;10:595. doi: 10.3389/fonc.2020.00595 (PMC7251179; doi:10.3389/fonc.2020.00595)

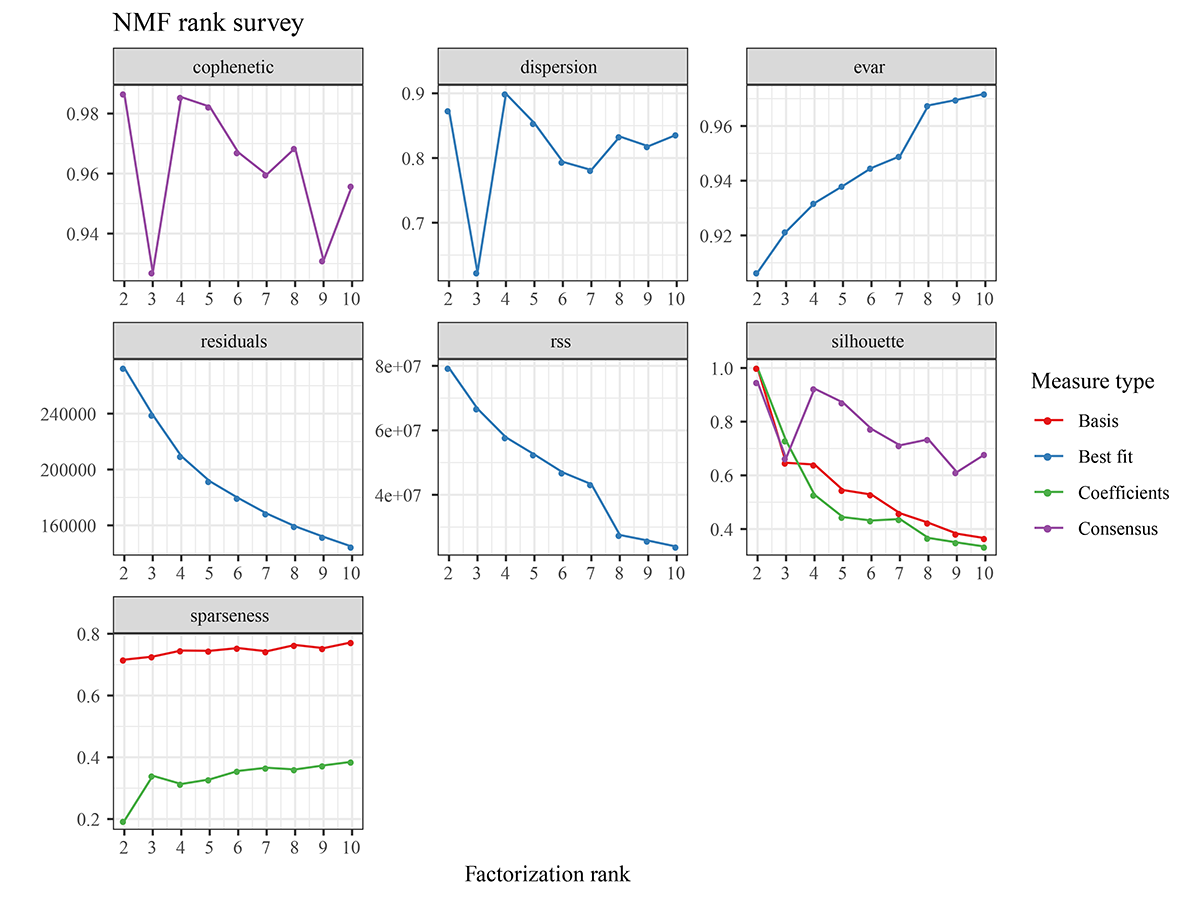

Supplement: Supplementary file 1 [file Data_Sheet_1.zip › Supplementary/Supplementary Figure1.tif]

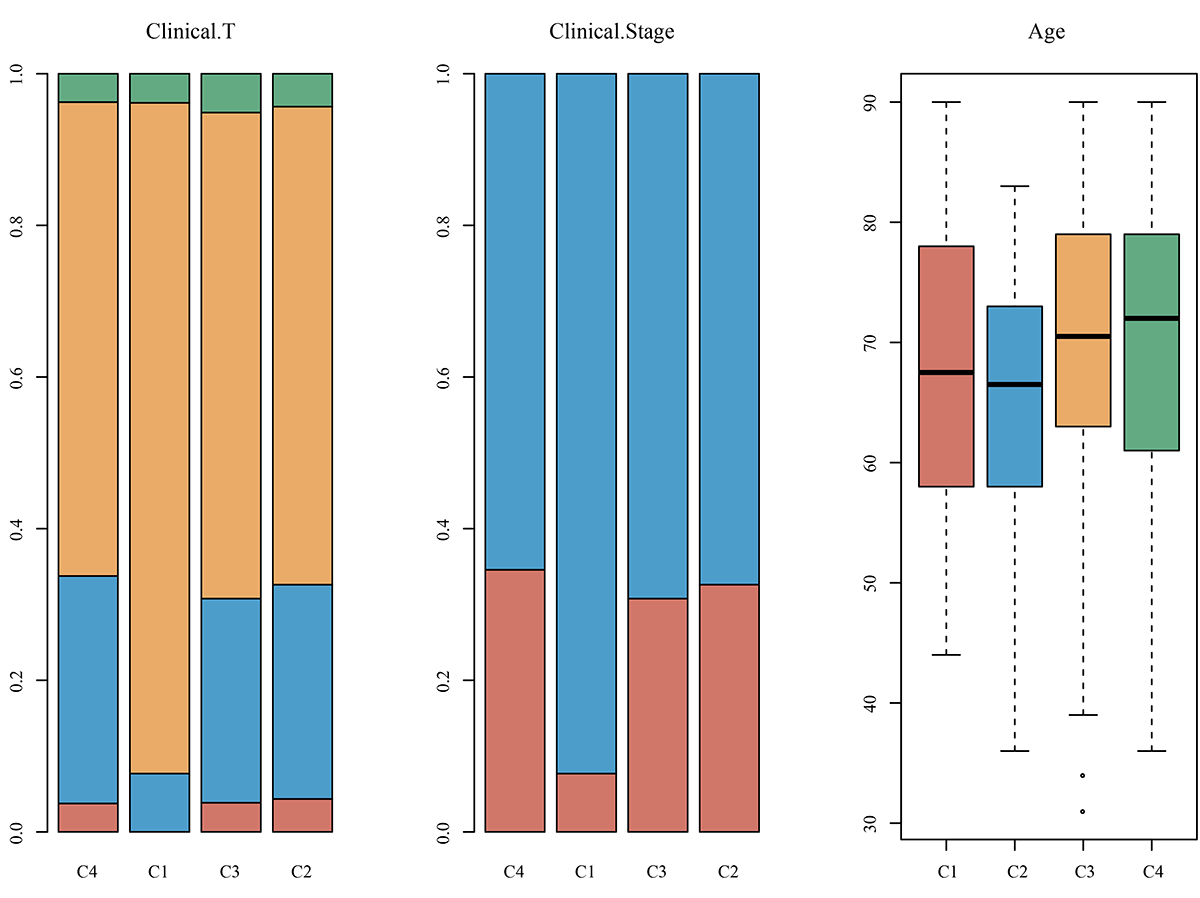

Supplement: Supplementary file 1 [file Data_Sheet_1.zip › Supplementary/Supplementary Figure2.tif]

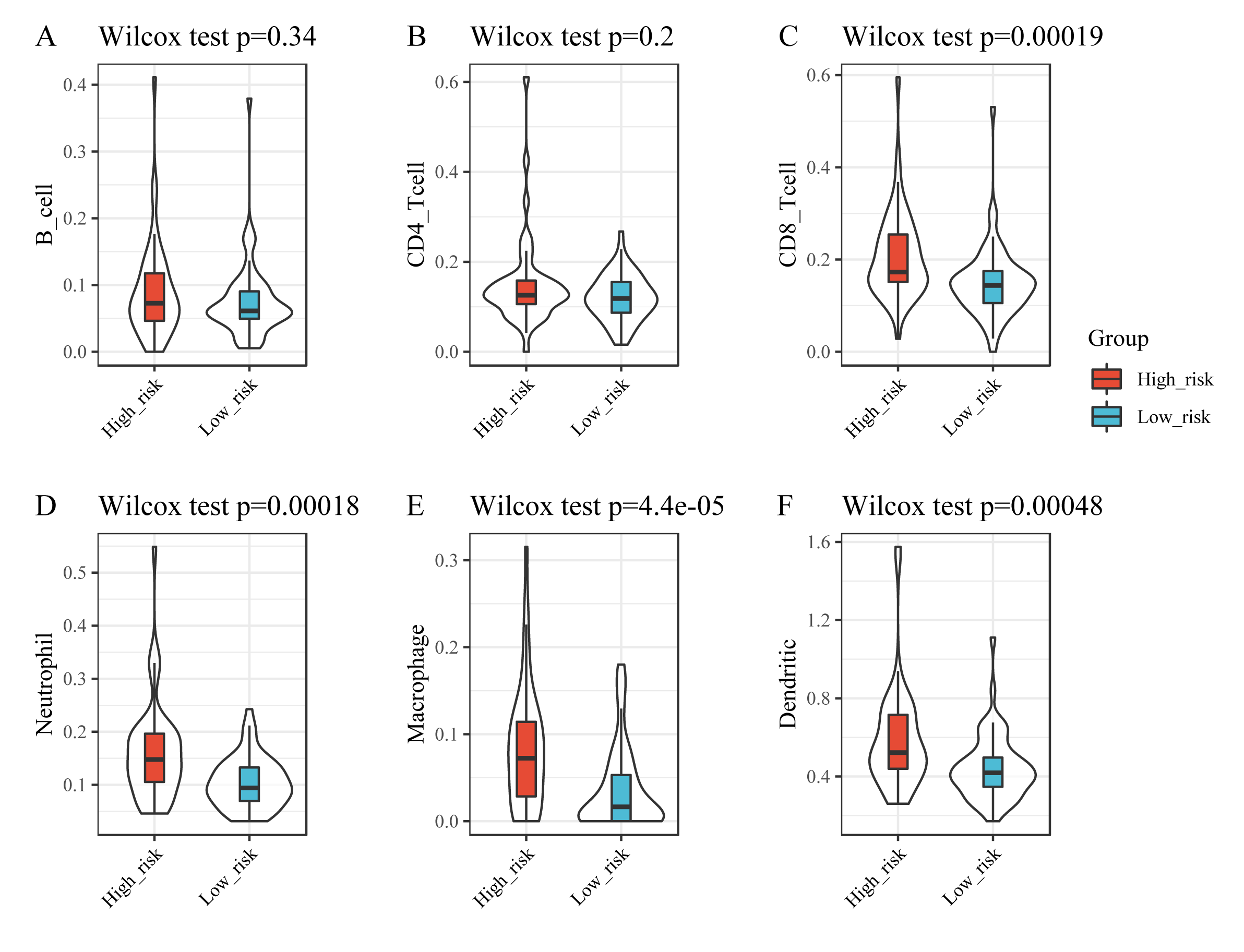

Supplement: Supplementary file 1 [file Data_Sheet_1.zip › Supplementary/Supplementary Figure3.tif]
